# Effect of cognitive retraining treatment in mild to moderate depressive disorders

**DOI:** 10.1186/s41155-023-00269-9

**Published:** 2023-09-18

**Authors:** Aarzoo Gupta, Santha Kumari

**Affiliations:** 1 Department of Psychiatry, Government Medical College & Hospital, Chandigarh, India; 2 School of Humanities & Social Sciences, Thapar Institute of Engineering & Technology, Patiala, India

**Keywords:** Depressive disorders, metacognition, dysfunctional beliefs, cognitive remediation, cognitive retraining

## Abstract

**Background:**

Cognitive retraining or remediation approaches dispense high levels of stimulation and new learning tasks, leading to an increased neural connections, which facilitate rapid recovery in patients with neurological as well as psychiatric conditions.

**Objectives:**

The current study aimed to investigate the effect of cognitive retraining (CR) in depressive disorders. We assigned 40 patients with mild to moderate depression to two sample groups, with 20 participants each: CR alone and CR with medicine. A 6-week CR module was delivered, and participants’ scores on measures such as the Beck Depression Inventory-II, Metacognition Questionnaire 30, World Health Organization Quality of Life- Brief, and Global Assessment of Functioning were compared.

**Results:**

Analysis using Stata/IC version 16 included descriptive statistics, paired and independent t-tests, analysis of covariance, and propensity score matching. Cohen's d was computed to determine the effect size. Within-group analysis revealed statistically significant differences in pre-post scores of the outcome measures (*p* < .05) and large effect size (*d* = 3.41; *d* = 3.60) in both groups. The difference in scores of outcome measures between the groups was not significant (*p* > .05) even when covariates were controlled, or nearest neighbor match analysis was carried out. CR is effective in alleviating symptoms and dysfunctional metacognitive beliefs in addition to enhancing functioning and quality of life.

**Conclusions:**

CR-based interventions may be essential mental health services owing to growing research in psychotherapy via virtual modes such as tele- and video-conferencing. These interventions can substantiate both prevention and remedy.

## Introduction

Depression is a common mood disorder, with an estimated 322 million globally and 57 million in India diagnosed with a depressive disorder (World Health Organization, 2017). Often depressive disorders are akin to low treatment adherence, relapse, and poor prognosis (World Health Organization, 1982). A vast majority (67–70%) of individuals are associated with disability due to mood disorders, as inter-episode recovery is characterized by residual symptoms (Mehta et al., 2014). Mood disorders are the leading cause of disability, that is, 13.4% of life years disability in women, and 8.3% in men (Üstün et al., 2004); and increased disability, which impacts multiple aspects of an individual's life, such as education, marriage, work, and social life, account for 4.3% of all disability-adjusted life years (DALYs) (Ferrari et al., 2013).

The meta-analysis showed significant correlations between depression severity and cognitive performance, although results concerning the affected cognitive domains are inconclusive (Bora et al., 2012). The cognitive domains reported to be involved include episodic memory, executive function, and processing speed (Bora et al., 2012; Hammar & Årdal, 2009; McDermott & Ebmeier, 2009). Patients with unipolar depression mainly exhibit cognitive inhibition deficits, problem-solving impairments, and planning deficits (Fossati et al., 2002). The previous body of work highlights the role of deficits in the poor functioning of patients and causing more regressions (Majer et al., 2004; Monkul et al., 2007). A depressed individual tends to center attention on the dysfunctional thoughts, reasoning about negative experiences, events, or emotions (Papageorgiou & Wells, 2003) breeding perseveration in the form of rumination and worry (Halvorsen et al., 2015). This perseverative thinking style or cognitive inflexibility is mediated by metacognitive beliefs (Jelinek et al., 2017). This omitted awareness and dysfunctional metacognitive beliefs impede daily functioning in the clinical population (Tajrishi et al., 2011). Wells (2009, p. 13–17) classified dysfunctional metacognitive beliefs operating through a cognitive attentional syndrome (CAS) and ascertained to be correlated with psychopathology. Positive and negative dysfunctional metacognitive beliefs lead to decreased self-esteem and increased affective symptoms (Kraft et al., 2017; Moses-Payne et al., 2019).

Multiple studies have proclaimed improvements in measures of functional capacity or functional outcome after cognitive retraining (Woolf et al., 2022). Cognitive remediation programs demonstrated efficiency in patients with brain lesions, and gradually these were extended to patients with schizophrenia, yielding significant improvements in cognitive performance, psychosocial functioning, and symptoms (Penadés & Catalán, 2012). The mechanisms of change employing cognitive retraining underlie brain plasticity, conceptualized as the potential of the brain to adapt and restore lost functions. Diller's descriptive model of cognition elucidates cognitive retraining precludes diagnosing the defect of particular ability and choosing a task that appeals to the respective ability adequately (Diamant & Hakkaart, 1989). The ability and task are then evaluated based on the activities of daily life (ADL), ensuing functional outcome, and its role in rehabilitation. Cognitive retraining aims at improving attention, memory, language, and/or executive functions utilizing a variety of manual or computerized exercises (Lampit et al., 2022; Woolf et al., 2022). These exercises aid in reducing cognitive deficits that often interfere with a person's ability to carry out routine activities, such as recalling faces or names of persons, attentive in conversation, and doing things. Cognitive retraining allows individuals with cognitive impairment to function productively and independently (Tomás et al., 2010).

Cognitive retraining therapies have produced advantageous developments in attention deficit hyperactivity disorder (Stevenson et al., 2002), learning disabilities, obsessive–compulsive disorders, and brain lesion patients, and many more (Buhlmann et al., 2006). Attempts have been made to enhance cognitive functioning in bipolar affective disorders, major depressive disorders, obsessive–compulsive disorders, anorexia nervosa, and substance use disorders (Lampit et al., 2022; Lee et al., 2013; Woolf et al., 2022). These trials imply that the performance of cognitive exercises or newly learned strategies could be practiced and generalized in ordinary behaviors.

We sought to replicate and extend extant findings by performing a comparative efficacy test of a Cognitive Retraining versus a Cognitive Retraining and Medication interventions. Escitalopram is a cost-effective and tolerable drug for treating mild to moderate severity of depression (Knorr et al., 2011; Murdoch & Keam, 2005; Skandali et al., 2018), though there are reported side effects associated with drop-outs. As an example, men tend to drop-out to avoid ejaculatory disorders (Li et al., 2017; Murdoch & Keam, 2005). Escitalopram as associated with improvement in verbal and visual memory in elderly patients suffering from stroke as well as depression when compared with other interventions (Jorge et al., 2010; Savaskan et al., 2008) various patients who walk-in the OPD do not prefer to seek psychotherapy due to barriers such as distance, time, and money. Medications alone is effective but adjunct is superior. In routine clinical practice, the researcher had found that the attention enhancing tasks, and simple arithmetic resulted in treatment adherence. Therefore, disseminating CRT may be an effective intervention for the clinical population who does not either prefer or access the traditional talk-therapies. This stirred the authors to study its effectiveness in comparison to the most commonly prescribed medication. The need to develop cognitive retraining-based interventions in depressive disorders has been highlighted in the existing literature with favorable denouements.

## Methods

### Design and setting

The current study examined the effect of cognitive retraining (CR) on symptom alleviation, metacognitive beliefs, quality of life, and global functioning in patients with depressive disorders. It was an intervention study utilizing within and between-group randomized design (Singh, 1998). A sample of 40 participants was recruited through probability sampling (Singh, 1998). Participants diagnosed with depressive disorders as per the ICD10 CDDG (World Health Organization, 1982) were recruited from the Behavior Therapy Unit of the out-patient Department of Psychiatry of a government tertiary care hospital located in an urban area. The patients referred to BT Unit were approached for consent and enrolled in the treatment groups, those receiving cognitive retraining alone (CRA) and those receiving cognitive retraining with medicine (CRM).

### Participants

**Fig. 1:**
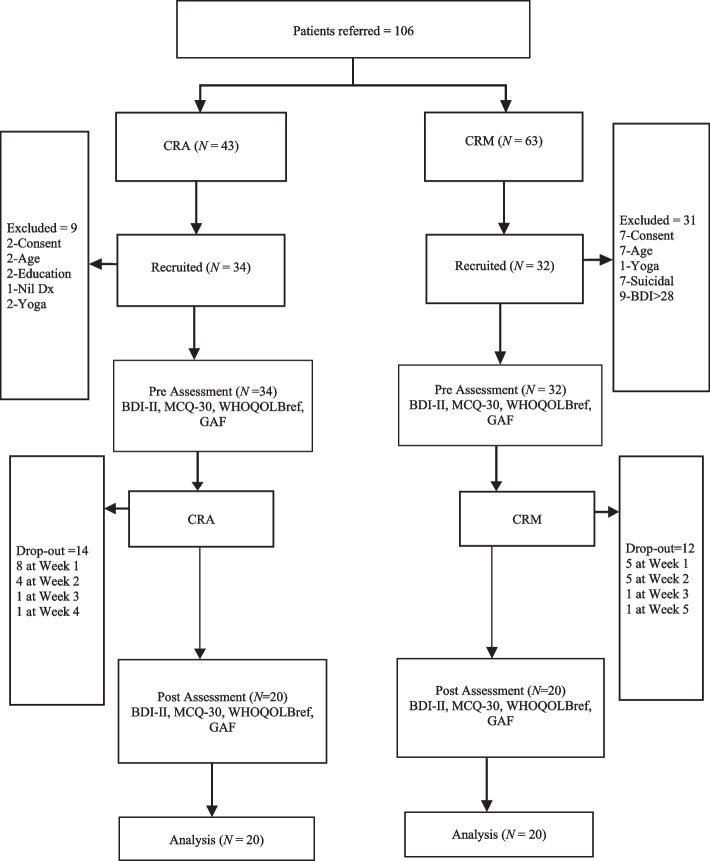
CONSORT diagram for participants throughout the study

Forty participants were enlisted in two groups as per the inclusion/exclusion criteria. Participants between 20 and 45 years of age of both sexes, with a minimum of 10 years of formal education and a clinical diagnosis of depressive disorders, were included. We excluded those with psychiatric comorbidity, severe depression, suicidality, clinical evidence of intellectual disability, suffering from any terminal illness, and neurological condition. As well, participants with a history of head injury, having received electroconvulsive therapy (ECT), any evidence-based psychotherapy, and practicing yoga/meditation/art of living by the time of the study or in the previous 6 months were excluded Fig. 1.

### Intervention

Cognitive retraining is a behavioral intervention that targets the neural systems through performance of certain paper–pencil tasks. These tasks stimulate the brain, acting as brain gym that restores the cognitive functions resulting in improved neural mechanisms producing the behavioral change (Keshavan et al., 2014). The tasks can aim various cognitive functions such as attention, memory, and executive functions etc. The tasks included in the 6-week module of cognitive retraining (CR) for depressive disorders were taken from the home-based CR module for schizophrenia developed for the Indian population by researchers at the National Institute of Mental Health and Neurosciences (NIMHANS; Hegde et al., 2012). The CR module used in the current study comprised 42 sessions spread over 6 weeks utilizing 112 total tasks as described in Table 1.

**Table 1:** Distribution of tasks of 6-week CR module

Week	Domain	Tasks	Task description
1	Attention (Attn)	Number Connection (NC)	Participant connects numbers (1–50) in a sequence which are randomly presented in space in a box on an A4 sheet. The numbers increase thru week 1 to 3
	Working Memory (WM)	Digit Sequencing (DS)	Two-digit numbers are presented and the participant is required to repeat immediately. The difficulty level has 2-digit numbers from 3 to 10
	Mental Speed (MS)	Letter Symbol Substitution (LSS)	An A4 sheet had boxes split in two parts, upper half has an alphabet and lower half was left empty for the participant to match the symbol for each alphabet, given in a row on top the sheet, and pen it down (5 rows)
2	Attn	NC	As above (1–75)
	WM	DS	As above (3–10)
	MS	LSS	As above (10 rows)
3	Attn	NC	As above (1–100)
	Information processing (IP)	Grain Sorting (GS)	The task requires the participant to sort 2 types of grains (Beans and split chickpeas), A 100-g amount of each grain was used
	WM	Calculation (Cal)	Ten numerical problems are solved using addition, subtraction, division, and multiplication
4	Attn	Letter Cancellation (LC)	Participant cancels 2 letters appearing among randomly presented English alphabets on an A4 sheet (42 rows, 53 columns)
	IP	GS	As above (green gram and rice)
	WM	Cal	As above (10 problems)
5	Attn	LC	As above (42 rows, 53 columns)
	Planning (Pl)	Mazes (Mz)	Participant moves through two mazes presented in square of 9.53 cm each without lifting pencil avoiding alleys
6	Attn	LC	As above (60 rows, 60 columns)
	Pl	Mz	As above

*CR *Cognitive retraining, *cm *Centimeters

### Outcome measures

The measures were applied in a face-to-face individual setting.

### Mini-International Neuropsychiatric Interview (MINI)

MINI is a measure of the 17 most common psychiatric disorders (Sheehan et al. 2016). The time taken for administration has a median of 26 min. MINI 7.0.2 is a revised version for both DSM-5 and ICD-10 diagnostic criteria. Inter-rater reliabilities of the MINI 7.0.2 ranged from 0.76 and 0.93 (Lecrubier et al., 1997). The English version was administered by the researcher.

### Beck Depression Inventory (BDI-II)

It is a 21-item measure of severity of depression. Participants respond on a 4-point Likert scale with scores ranging from 0 to 3 (Beck et al., 1996). It takes 5–10 min to complete BDI-II (Farinde, 2013). Previous studies reported internal consistency of 0.09 and retest reliability of 0.73 to 0.96 (Wang & Gorenstein, 2013). The printed copy of BDI-II (English version) was provided and the participant chose response that explained his symptom the best.

### The Metacognitive Questionnaire (MCQ-30)

MCQ30 assesses the metacognitive mode of psychological disorders (Wells, 2009). It includes 30 items rated on a 4-point Likert scale (from 1 to 4). It has five sub-scales and takes around 25 to 30 min to complete. The internal consistency of the total score from this measure ranged from 0.72 to 0.93 (Wells and Cartwright-Hatton, 2004). The participant was given printed copy of the questionnaire in English and asked to choose response applied to him.

### World Health Organization Quality of Life-Brief (WHOQOLBref)

It is a 26-item with good discriminant validity, content validity, and test–retest reliability of Hindi version (Saxena et al., 1998). It enquires about the quality of life in the last 2 weeks on 4 domains. Each item is rated on a 5-point scale (0–5) and takes only 5–8 min to complete. Cronbach’s alpha values for scores from the WHOQOLBref were 0.85 for psychological health, 0.73 for physical health, 0.73 for social relationships, and 0.68 for the environment (Oliveria et al. 2016). The participant responded quality of their life in various domains on the Hindi version copy of the scale.

### Global Assessment of Functioning (GAF)

The GAF is a generic measure of how a patient is doing (Kaplan & Sadock, 1998). Inter-rater reliability scores range from 0.39 to 0.59 while among researchers was from 0.81 to 0.85 (Vatnaland et al., 2007). The GAF score was marked by the researcher based on enquiry of personal, social, and occupational functioning.

### Procedure

The Ethics Committee of the Institute approved the study (GMCH/IEC/2019/316). The psychiatrist referred the patients for psychological intervention from the outpatient department of Psychiatry. Every consecutive patient with depressive disorder referred to the Behavior Therapy Unit of the Department was approached for the consent as per Declaration of Helsinki (Williams, 2008). Those who consented, their socio-demographic and clinical details were recorded by the researcher using a performa developed in accordance with the inclusion and exclusion criteria. Those who fulfilled the criteria of a major depressive disorder (MDD) or recurrent depressive disorder (RDD) as per MINI 7.0.2 were included, and the severity of their depression was evaluated using the BDI-II. Those who scored between and 14–28 on BDI-II, suggesting mild to moderate depression were included (Smarr & Keefer, 2011). The recruited participants linked for psychotherapy were assigned to the treatment groups as per their prescription; those who had not been prescribed any medicine were assigned to the CRA group, whereas that prescribed medicine (only one selective serotonin reuptake inhibitor) were assigned to the CRM group. The assessment of each participant was conducted before initiating the intervention on the outcome measures: MCQ30, WHOQOLBref (QOL), and GAF.

The completion of the assessment was followed by initiating CR for all participants in both the groups. The intervention (CR) was introduced to each participant with a standard set of instructions emphasizing the importance of improved brain functioning in reducing the symptoms. Further, the process of weekly sessions and performing the tasks at home monitored by a family member were explained. A face-to-face session was scheduled every 7th day as progress was made to the next module. In this manner, all six modules were delivered. The incentive offered to the participants was assistance in OPD registration and instant psychiatry consultation after bypassing the queue for next 6 months and to those only coming for CR, assistance to any other OPD of the Hospital for 3 months was offered. The assessment was repeated on the outcome measures (BDI-II, MCQ30, QOL, GAF) after the completion of delivery of the intervention module. The study was terminated, and after that, the participants in both groups continued to seek the required services from the OPD of Psychiatry.

### Statistical analysis

The quantified data were analyzed using the software for statistics and data science Stata/IC version 16. Descriptive statistics, paired t-test and independent t-test was computed. Further, we used analysis of covariance (ANCOVA) to control confounding variables such as diagnosis, number of episodes of depression, and duration of illness. Propensity score matching was employed to overcome the limitation of purposive sampling by comparing each case to its nearest neighbor match (Austin, 2011). Cohen’s *d* was also used to determine the effect size of these changes in response to the intervention (Sawilowsky, 2009).

## Results

The participants in the two groups showed no significant differences in age, sex, or education (*p* > 0.05). There was equal distribution of those having diagnosis of MDD and RDD in both groups. The CRM sample (46.3 ± 71.78) had a greater mean duration of illness than the CRA sample (33.85 ± 36.24), implying the need of psychiatrist prescribed anti-depressant medicine (ADM) to the CRM sample. There was compliance with the task performance indicating the feasibility and convenience of CR as an independent as well as an adjunct. The compliance to CR intervention in both the treatment groups was more than 80% with mean of 111 and 110 tasks completed by the participants in CRA and CRM groups respectively (Table 2).

**Table 2:** Characteristics of participants in the two treatment groups (CRA and CRM)

Variables	CRA	CRM	*p*
Age *M *(SD)	27.1 (6.45)	30.35 (9.50)	.213
Education *M *(SD)	15.4 (1.79)	14.65 (2.11)	.232
Sex *f (%)*			
Female	11 (55)	10 (50)	.752
Male	9 (45)	10 (50)	
Diagnosis *f (%)*			
MDD	11 (55)	11 (55)	1.000
RDD	9 (45)	9 (45)	
Episodes* f (%)*			.890
0	11 (55)	11 (55)	
1	6 (30)	5 (25)	
2	3 (15)	4 (20)	
DOI (Months)* M *(SD)	33.85 (36.24)	46.3 (71.78)	.492
Number of sessions *M *(SD)	111.05 (2.37)	109.75 (4.59)	.267

*CRA *Cognitive retraining alone, *CRM *Cognitive retraining with medicine, *M *Mean, *SD *Standard deviation, *f *Frequency, % Percentage, *MDD *Major depressive disorder, *RDD R*ecurrent depressive disorder, *DOI *Duration of illness

^*^p < .05. **p < .01. ***p < .001

The paired t-test analysis (Table 3) revealed a statistically significant difference in pre-treatment (assessment carried out before starting of CR) and post-treatment (assessment carried out after delivery of 6-week CR module) scores of the outcome measures (BDI-II, MCQ30, QOL, GAF) wherein there was a decrease in symptoms (BDI-II) in both samples (*p* < 0.001). The effect size was in the same range for both treatment groups (*d* = 3.41; *d* = 3.60), denoting a substantial effect. It was observed that change was more extensive in dysfunctional positive beliefs (*d* = 1.45) in the CRA sample, whereas in the CRM sample, Cohen's *d* was more significant (*d* = 2.15) in dysfunctional negative beliefs. The post-assessment scores on scales of functioning (QOL and GAF) increased significantly (*p* < 0.01) in both the samples when compared with the pre-assessment scores. The improvement varied among domains of functioning, and more significant effect size (Cohen’s *d*) was observed in the CRM group when the intervention was combined with the medicine except for the social relations of QOL.

**Table 3:** Within group differences on outcome measures before (pre-treatment) and after (post- treatment) the intervention

	CRA (*df* = 19)						
Measures	Pre-treatment		Post-treatment		*t*	*p*	*d*
	*M*	SD	*M*	SD			
BDI-II	23.9	4.24	6.8	5.68	16.97	.000	3.41
MCQ30							
Total	65.65	18.17	43.45	12.87	6.30	.000	1.41
POS	12.45	3.36	8.35	2.16	5.90	.000	1.45
NEG	16.05	4.67	10.1	5.01	5.56	.000	1.23
CC	10.55	4.27	8.00	3.46	3.42	.002	.65
NC	13.6	4.88	8.35	3.73	4.62	.000	1.21
CSC	12.75	4.40	10.05	3.82	3.26	.004	.65
QOL							
PH	20.35	3.34	26.00	4.04	4.45	.000	1.52
PSY	13.6	3.01	19.9	3.11	5.64	.000	2.06
SR	7.4	2.09	9.55	1.50	4.56	.000	1.18
ENV	23.75	3.77	25.75	3.77	2.94	.008	.53
GAF	55.9	7.97	68.45	9.58	9.90	.000	1.42
	CRM						
Measures	Pre-treatment		Post-treatment		*t*	*p*	*d*
	*M*	SD	*M*	SD			
BDI-II	22.4	5.03	5.00	4.61	14.30	.000	3.60
MCQ30							
Total	63.85	14.61	44.8	10.50	5.54	.000	1.51
POS	10.1	3.81	8.00	2.34	3.30	.003	.66
NEG	16.25	4.20	9.2	1.96	9.24	.000	2.15
CC	10.35	3.08	7.3	2.32	3.78	.001	1.12
NC	13.25	4.05	8.85	3.67	4.09	.000	1.14
CSC	13.6	4.40	11.1	4.05	2.26	.035	.60
QOL							
PH	19.15	5.02	26.55	4.04	8.43	.000	1.62
PSY	15.4	4.34	21.5	3.07	8.02	.000	1.62
SR	8.6	3.36	10.85	2.43	6.48	.000	.77
ENV	24.85	6.90	27.85	6.28	3.39	.003	.45
GAF	57.55	4.76	73.75	6.19	14.08	.000	2.93

*CRA* Cognitive retraining alone, *CRM* Cognitive retraining with medicine, *M* Mean, *SD* Standard deviation, *BDI-II* Beck depression inventory, *MCQ30* Metacognition questionnaire, *POS* Positive belief about worry, *NEG* Negative beliefs about uncontrollability and danger of worry, *CC* Cognitive confidence, *NC* Need for control, *CSC* Cognitive self-consciousness, *QOL* World Health Organization quality of life brief, *PH* Physical, *PSY* Psychological, *SR* Social relations, *ENV* Environmental, *GAF* Global assessment of functioning

*p < .05. **p < .01. ***p < .001

The independent *t* test analysis (Table 4) revealed that the difference in scores of outcome measures between the two samples was not significant (*p* > 0.05) except for SR (*p* = 0.049; *p* < 0.05) and GAF (*p* = 0.044; *p* < 0.05). This difference suggests that both CRA and CRM was effective treatment options for this sample of depressive disorders. The effect size (*d*) for between-group differences ranged from small to medium. There was no significant difference (*p* > 0.05) observed even when covariates such as diagnosis, episodes, and duration of illness were controlled (Table 5), except for global functioning (*p* < 0.001). The participants in the CRA sample were compared with the CRM sample with its nearest match based on diagnosis, episodes, duration of illness, and pre-treatment scores of each outcome measure on every domain and sub-domains. Even after the nearest neighbor match (NNM) analysis, no significant difference was observed in the post-treatment scores of outcome measures of the two groups, except for specific domains of quality of life (QOL), that is, SR (*p* = 0.029; *p* < 0.05) and ENV (*p* = 0.057; *p* < 0.05). This observation established that CR could be an effective intervention and may be used as a distinct treatment delivered independently or in combination with pharmacotherapy in depressive disorders.

**Table 4:** Comparing differences between groups (CRA vs CRM) on outcome measures

Measures	CRA *M *(SD)	CRM *M* (SD)	Mean difference	Standard error difference	*t*	*p*	*d*
BDI-II	6.8 (5.68)	5.00 (4.61)	1.8	1.64	1.10	.278	.35
MCQ30							
Total	43.45 (12.87)	44.8 (10.50)	1.35	3.71	.36	.718	.11
POS	8.35 (2.16)	8.00 (2.34)	.35	.71	.50	.626	.15
NEG	10.1 (5.01)	9.2 (1.96)	.9	1.20	.75	.459	.24
CC	8.00 (3.46)	7.3 (2.32)	.7	.93	.75	.457	.24
NC	8.35 (3.73)	8.85 (3.67)	.5	1.17	.43	.672	.13
CSC	10.05 (3.82)	11.1 (4.05)	1.05	1.24	.84	.404	.27
QOL							
PH	26.00 (4.04)	26.55 (4.04)	.55	1.28	.43	.669	.14
PSY	19.9 (3.11)	21.5 (3.07)	1.6	.98	1.64	.109	.52
SR	9.55 (1.50)	10.85 (2.43)	1.3	.64	2.03	.049*	.64
ENV	25.75 (3.77)	27.85 (6.28)	2.1	1.64	1.28	.207	.40
GAF	68.45 (9.58)	73.75 (6.19)	5.3	2.55	2.08	.044*	.66

*CRA* Cognitive retraining alone, *CRM* Cognitive retraining with medicine, *M* Mean, *SD* Standard deviation, *BDI-II* Beck depression inventory, *MCQ30* Metacognition questionnaire, *POS* Positive belief about worry, *NEG* Negative beliefs about uncontrollability and danger of worry, *CC* Cognitive confidence, *NC* Need for control, *CSC* Cognitive self-consciousness, *QOL* World Health Organization quality of life brief, *PH* Physical, *PSY* Psychological, *SR* Social relations, *ENV* Environmental, *GAF* Global assessment of functioning

^*^p < .05. **p < .01. ***p < .001

**Table 5:** Comparing differences between groups (CRA vs CRM) on outcome measures after controlling confounding variables

Measures	ANCOVA			PSM-NNM		
	*F*	*R*^2^	*p*	*Coef*	*z*	*p*
BDI-II	.49	.05	.739	1.72	1.26	.208
MCQ						
Total	.91	.09	.468	.69	.17	.864
POS	.45	.05	.772	.43	.41	.683
NEG	1.04	.11	.402	2.09	1.30	.195
CC	.59	.06	.674	1.07	1.29	.197
NC	.95	.10	.445	.14	.11	.914
CSC	1.63	.16	.189	.75	.60	.551
QOL						
PH	1.15	.12	.351	.1	.11	.915
PSY	1.17	.12	.340	1.51	1.57	.117
SR	2.14	.20	.096	1.17	2.18	.029**
ENV	.61	.06	.659	2.64	1.90	.057
GAF	5.96	.40	.000***	2.35	1.10	.272

*CRA* Cognitive retraining alone, *CRM* Cognitive retraining with medicine, *PSM* Propensity score matching, *ANCOVA* Analysis of covariance, *BDI-II* Beck depression inventory, *MCQ30* Metacognition questionnaire, *POS* Positive belief about worry, *NEG* Negative beliefs about uncontrollability and danger of worry, *CC* Cognitive confidence, *NC* Need for control, *CSC* Cognitive self-consciousness, *QOL* World Health Organization quality of life brief, *PH* Physical, *PSY* Psychological, *SR* Social relations, *ENV* Environmental, *GAF* Global assessment of functioning

^*^p < .05. **p < .01. ***p < .001

## Discussion

The findings of the present study concluded that CR was effective in alleviating symptoms and dysfunctional metacognitive beliefs as well as enhancing functioning and quality of life. Within-group analysis displayed a significant effect on all outcome measures with its corresponding effect size. Between-group comparisons revealed no significant difference in outcome measures, except for a few domains of metacognitive beliefs and quality of life. The outcome of both treatment options produced no significant difference even when controlling for confounding variables using ANCOVA and PSM analysis. This outcome implied that cognitive retraining was effective in depressive disorders sample of the study and may be disseminated as a discrete form of treatment or combined with pharmacotherapy. Cognitive retraining programs remediate attention, the most elementary cognitive function, plus executive function, visuospatial learning, and memory (Kennedy et al., 2007) crucial to full functional recovery. Attention allows greater cognitive energy for information processing and amplifies metacognitive awareness, contributing to curtail ruminative thinking and extended cognitive flexibility. Positron emission tomography (PET) and functional magnetic resonance imaging (fMRI) studies have shown that ruminative thinking reduces prefrontal cortex activities that devolve day-to-day functioning due to contrived problem solving and decision making (DeRubeis et al., 2008). Lowered metacognitive awareness validates dysfunctional metacognitive beliefs and reduces cognitive flexibility. Therefore, CR might be considered as an effective behavioral technique that sharpens cognitive processing (Porter et al., 2013) and demotes ruminative thinking. Hence, interventions targeting the reduction of ruminations favor enhanced cognitive flexibility, which improves psychosocial functioning. Traditional cognitive approaches focus on challenging, disputing, or replacing ruminations or depressive cognitions, wherein third-wave therapies promote the use of mindfulness-based practices accelerating awareness of here and now. These interventions boost awareness, complementing psychosocial functioning, and alleviation of symptoms such as cognitive retraining. The difference is in mechanisms; cognitive-behavioral approaches debate cognitive, affective, and/or conative (CAC) patterns that revamp cerebral structure and neuropsychological functioning. On the other hand, cognitive retraining concentrates on underlying neurobiological mechanisms prompting changes in CAC patterns (Fergus & Bardeen, 2016). CR-based interventions have been aptly used in OCD, anorexia nervosa, bipolar affective disorders, neurotic disorders, etc. (Kim et al., 2018). Cognitive deficits in these disorders include verbal fluency, executive function, working memory, retention; and CR-based interventions lead to improved cognitive functioning with moderate to large effect size (McGurk et al., 2007). More recently, it has been studied in those with intellectual disabilities to ameliorate cognitive processes (García-Alba et al., 2020). The authors’ affiliated department has developed a software-based cognitive enhancement program for those with severe mental illnesses (Singh et al., 2023). The first author also has attempted to use the CR module as an adjunct dealing with non-compliance in cognitive-behavioral or talk-therapies, and the results have been promising (Kashyap & Gupta, 2022). Miscellaneous versions of cognitive retraining techniques have been examined, yet evidence-based standardized modules are countable. The tasks and length of program diverge, a multimodal behavioral intervention program of 10 days, 4 h per day with a post-intervention follow-up at 6, 12, and 18 months induced higher functional abilities in patients with mild cognitive impairment (MCI) (Amofa et al., 2020). A novel virtual reality (VR)-based program combining aerobic exercise and cognitive training has been evaluated in the elderly population (> 65 years) with MCI and Alzheimer’s disease (AD). No statistical significance was obtained when comparing within and between both groups due to the small sample size. Still, self-perceived improvement performance in real life was fostered in VR-based training of 6 weeks, three sessions/week (18 sessions total) lasting approximately 40–45 min long combining physical and cognitive training (Mrakic-Sposta et al., 2018). The attention training technique (ATT) used by Wells is comparable to any cognitive retraining intervention (Fergus & Bardeen, 2016). Siegel states that ATT is a neurobiological therapy that bourns biological mechanisms underlying psychological disorders. CAS, central to S-REF (self-regulatory executive function), breeds excessive processing of threat in the form of worry and rumination, consequently hindering attentional control. Cognitive retraining interventions tend to modify the focus of attention that mitigates CAS, meaning that discounted cognitive energy in the processing of certain beliefs contributes to cognitive flexibility and improved cognitive functioning. A systematic review of cognitive interventions for depressive disorders disport varied interventions as brief as single-session manipulation to daily online sessions for 10 days as intense as 36 sessions for 12 weeks have laid out anticipated consequences (Koster et al., 2017). Likewise, refined neurobiological functioning proclaimed via psychosocial functioning. CR in depressive disorders has recently grown after acknowledging the deficits present even in the euthymic state, and the growing prevalence of depression, the associated disability may be prevented using CR-based interventions by increasing cognitive functioning.

### Limitations and future research

The limitation of the present study was the absence of longitudinal follow-up. However, a few study participants, who continued seeking clinical services in the OPD after the termination of the study, reported that CR tasks helped them evolve persistence, optimism, and confidence. It would have been beneficial to use standardized measures of rumination, attention, or neurocognitive function as outcome measures. The addition of any other outcome measure would have resulted in increased time per participant which usually is not preferred by the patients visiting the OPD. Demographic variables and clinical variables were not matched; nevertheless, confounding variables were statistically handled. Similarly, nonprobability sampling and non-randomized assignment to treatment groups were also settled statistically using propensity score matching analysis. Lastly, a pre-post design limited the robustness of methodology, having a third-group of those receiving only medication would have led to better comparison of the findings. However, future studies can be planned with more robust methodology and addressing above limitations.

## Conclusion

The authors have tried to propose CR may be a potent treatment option for depressive disorders. CR-based interventions are free from the use of metaphors and stimuli, unlike cognitive-behavioral approaches; therefore, these might more convenient and probably culture-free. CR might be superior in rendering services to patients coming from geographically distant or remote areas requiring fewer sessions with specialists. This perception may endorse the vision of the World Health Organization (WHO) on mental health policy and service provision (World Health Organization 2001) to train and involve non-specialists in augmenting the mental health service delivery system (Mendenhall et al., 2014). In conclusion, CR-based interventions may contribute in prevention as well as remedy.

## Data Availability

The data can be provided to the Editorial Team on request.

## Abbreviations

AD: Alzheimer’s disease
ADL: Activities of daily living
ADM: Anti-Depressant Medicine
ANCOVA: Analysis of covariance
ATT: Attention training technique
BDI-II: Beck Depression Inventory
CAC: Cognitive, affective, conative
CAS: Cognitive attentional syndrome
CC: Cognitive confidence
CR: Cognitive retraining
CRA: Cognitive retraining alone
CRM: Cognitive retraining with medicine
CSC: Cognitive self-consciousness
ECT: Electroconvulsive therapy
ENV: Environmental
fMRI: Functional magnetic resonance imaging
GAF: Global assessment of functioning
GMCH: Government medical college and hospital
ICD10 CDDG: International Classification of Diseases, Tenth Revision: Clinical Descriptions and Diagnostic Guidelines (CDDG)
IEC: Institution Ethics Committee
MCI: Mild cognitive impairment
MCQ-30: The Metacognitive Questionnaire
MDD: Major depressive disorder
MINI: Mini-International Neuropsychiatric Interview
NC: Need for control
NEG: Negative Beliefs about Uncontrollability and Danger of Worry
NIMHANS: National Institute of Mental Health and Neurosciences
NNM: Nearest neighbor match
OCD: Obsessive-compulsive disorder
OPD: Out-patient department
PET: Positron emission tomography
PH: Physical
POS: Positive Belief About Worry
PSM: Propensity Score Matching
PSY: Psychological
QOL/WHOQOLBref: World Health Organization Quality of Life-Brief
QOL: World Health Organization Quality of Life Brief,
RDD: Recurrent depressive disorder
SR: Social relations
VR: Virtual reality
WHO: World Health Organization

## References

[CR1] Amofa PA, DeFeis B, De Wit L, O’Shea D, Mejia A, Chandler M, Locke DEC, Fields J, Phatak V, Dean PM, Crook J, Smith G (2020). Functional ability is associated with higher adherence to behavioral interventions in mild cognitive impairment. Clinical Neuropsychologist.

[CR2] Austin PC (2011). An introduction to propensity score methods for reducing the effects of confounding in observational studies. Multivariate Behavioral Research.

[CR3] Beck, A., Steer, R., & Brown, G. (1996). *Manual for the Beck Depression Inventory II*. Psychological Corporation.

[CR4] Bora E, Harrison BJ, Yücel M, Pantelis C (2012). Cognitive impairment in euthymic major depressive disorder: A meta-analysis. Psychological Medicine.

[CR5] Buhlmann U, Deckersbach T, Engelhard I, Cook LM, Rauch SL, Kathmann N, Wilhelm S, Savage CR (2006). Cognitive retraining for organizational impairment in obsessive-compulsive disorder. Psychiatry Research.

[CR6] DeRubeis, R. J., Siegle, G. J., & Hollon, S. D. (2008). Cognitive therapy versus medications for depression: Treatment outcomes and neural mechanisms. *Nature Reviews Nueroscience*, *9*(10), 788–796. 10.1038/nrn2345 10.1038/nrn2345 PMC2748674 18784657

[CR7] Diamant, J. J., & Hakkaart, P. J. W. (1989). Cognitive rehabilitation in an information-processing perspective. https://www.semanticscholar.org/paper/Cognitive-Rehabilitation-in-an-Perspective-Diamant-Hakkaart/eb163fea633d75ddfe03fd55b47158e0402255d0. Accessed 29 Jul 2020.

[CR8] Farinde A (2013). The Beck depression inventory. The Pharma Innovation International Journal.

[CR9] Fergus TA, Bardeen JR (2016). The attention training technique: A review of a neurobehavioral therapy for emotional disorders. Cognitive and Behavioral Practice.

[CR10] Ferrari, A. J., Charlson, F. J., Norman, R. E., Patten, S. B., Freedman, G., Murray, C. J. L., Vos, T., & Whitefield, H. A. (2013). Burden of depressive disorders by country, sex, age, and year: findings from the global burden of disease study 2010. *PLoS Medicine, 10*(11), e1001547. 10.1371/journal.pmed.1001547 10.1371/journal.pmed.1001547 PMC3818162 24223526

[CR11] Fossati P, Ergis AM, Allilaire JF (2002). Executive functions in unipolar depression: A review. L’encéphale.

[CR12] García-Alba J, Rubio-Valdehita S, Sánchez MJ, García AIM, Esteba-Castillo S, Gómez-Caminero M (2020). Cognitive training in adults with intellectual disability: Pilot study applying a cognitive tele-rehabilitation program. International Journal of Developmental Disabilities.

[CR13] Halvorsen M, Hagen R, Hjemdal O, Eriksen MS, Sørli ÅJ, Waterloo K, Eisemann M, Wang CEA (2015). Metacognition and thought control strategies in unipolar major depression: A comparison of currently depressed, previously depressed, and never-depressed individuals. Cognitive Therapy and Research.

[CR14] Hammar Å, Årdal G (2009). Cognitive functioning in major depression-a summary. Frontiers in Human Neuroscience.

[CR15] Hegde S, Rao SL, Raguram A, Gangadhar BN (2012). Addition of home-based cognitive retraining to treatment as usual in first episode schizophrenia patients: A randomized controlled study. Indian Journal of Psychiatry.

[CR16] Jelinek L, Quaquebeke NV, Moritz S (2017). Cognitive and metacognitive mechanisms of change in metacognitive training for depression. Scientific Reports.

[CR17] Jorge RE, Acion L, Moser D, Adams HP, Robinson RG (2010). Escitalopram and enhancement of cognitive recovery following stroke. Archives of General Psychiatry.

[CR18] Kaplan HI, Sadock BJ (1998). Kaplan & Sadock’s Synopsis of Psychiatry: Behavioural Sciences/Clinical Psychiatry.

[CR19] Kashyap A, Gupta A (2022). Cognitive retraining as adjunct toamange non-adherence in delivery of dialectical behaviour therapy: A case series. Delhi Psychiatry Journal.

[CR20] Kennedy N, Foy K, Sherazi R, Mc Donough M, Mc Keon P (2007). Long-term social functioning after depression treated by psychiatrists: A review. Bipolar Disorders.

[CR21] Keshavan MS, Vinogradov S, Rumsey J, Sherrill J, Wagner A (2014). Cognitive training in mental disorders: Update and future directions. American Journal of Psychiatry.

[CR22] Kim EJ, Bahk Y-C, Oh H, Lee W-H, Lee J-S, Choi K-H (2018). Current status of cognitive remediation for psychiatric disorders: A review. Frontiers in Psychiatry.

[CR23] Knorr U, Vinberg M, Gade A, Winkel P, Gluud C, Wetterslev J, Gether U, Kessing L (2011). A randomized trial of the effect of escitalopram versus placebo on cognitive function in healthy first-degree relatives of patients with depression. Therapeutic Advances in Psychopharmacology.

[CR24] Koster EHW, Hoorelbeke K, Onraedt T, Owens M, Derakshan N (2017). Cognitive control interventions for depression: A systematic review of findings from training studies. Clinical Psychology Review.

[CR25] Kraft B, Jonassen R, Stiles TC, Landrø NI (2017). Dysfunctional metacognitive beliefs are associated with decreased executive control. Frontiers in Psychology.

[CR26] Lampit A, Launder NH, Minkov R, Rollini A, Davey CG, Finke C, Lautenschlager NT, Gavelin HM (2022). Computerised cognitive training in people with depression: A protocol for a systematic review and meta-analysis. Systematic Reviews.

[CR27] Lecrubier Y, Sheehan DV, Weiller E, Amorim P, Bonora I, Sheehan KH, Janvs J, Dunbar GC (1997). The mini international neuropsychiatric interview (MINI). A short diagnostic structured interview: Reliability and validity according to the CIDI. European Psychiatry.

[CR28] Lee RSC, Redoblado-Hodge MA, Naismith SL, Hermens DF, Porter MA, Hickie B (2013). Cognitive remediation improves memory and psychosocial functioning in first-episode psychiatric patients. Psychological Medicine.

[CR29] Li, G., Shen, Y., Luo, J., & Li, H. (2017). Efficacy of escitalopram monotherapy in the treatment of major depressive disorder: A pooled analysis of 4 Chinese clinical trials. *Medicine*, *96*(39), e8142. 10.1097/MD.0000000000008142 10.1097/MD.0000000000008142 PMC5626292 28953649

[CR30] Majer M, Ising M, Künzel H, Binder EB, Holsboer F, Modell S, Zihl J (2004). Impaired divided attention predicts delayed response and risk to relapse in subjects with depressive disorders. Psychological Medicine.

[CR31] McDermott LM, Ebmeier KP (2009). A meta-analysis of depression severity and cognitive function. Journal of Affective Disorders.

[CR32] McGurk SR, Twamley EW, Sitzer DI, Mc Hugo GJ, Mueser KT (2007). A meta-analysis of cognitive remediation in schizophrenia. American Journal of Psychiatry.

[CR33] Mehta, S., Mittal, P. K., & Swami, M. K. (2014). Psychosocial functioning in depressive patients: a comparative study between major depressive disorder and bipolar affective disorder. *Depression Research and Treatment*, *2014*, 302741. 10.1155/2014/302741 10.1155/2014/302741 PMC3972948 24744917

[CR34] Mendenhall E, De Silva MJ, Hanlon C, Peterson I, Shidhaye R, Jordans M, Luitel N, Ssebunnya J, Fekadu A, Patel V, Tomlinson M, Lund C (2014). Acceptability and feasibility of using non-specialist health workers to deliver mental health care: Stakeholders perceptions form the PRIME district sites in Ethiopia, India, Nepal, South Africa, and Uganda. Social Science & Medicine.

[CR35] Monkul E, Green M, Barrett J, Robinson J, Velligan D, Glahn D (2007). A social cognitive approach to emotional intensity judgment deficits in schizophrenia. Schizophrenia Research.

[CR36] Moses-Payne ME, Rollwage M, Fleming SM, Roiser JP (2019). Post decision evidence integration and depressive symptoms. Frontiers in Psychiatry.

[CR37] Mrakic-Sposta S, Di Santo SG, Franchini F, Arlati S, Zangiacomi A, Greci L, Moretti S, Jesuthasan N, Marzorati M, Rizzo G, Sacco M, Vezzoli A (2018). Effects of combined physical and cognitive virtual reality-based training on cognitive impairment and oxidative stress in MCI patients: A pilot study. Frontiers in Aging Neuroscience.

[CR38] Murdoch D, Keam SJ (2005). Escitalopram. Drugs.

[CR39] Oliveira SHE, Carvalho H, Esteves F (2016). Toward understanding of the quality of life construct: Validity and reliability of the WHOQOL-Bref in a psychiatry sample. Psychiatry Research.

[CR40] Papageorgiou C, Wells A (2003). An empirical test of a clinical metacognitive model of rumination and depression. Cognitive Therapy and Research.

[CR41] Penadés, R., & Catalán, R. (2012). Cognitive remediation therapy (CRT): improving neuro cognition and functioning in schizophrenia. *Schizophrenia in the 21*^*st*^* Century*. 10.5772/36783

[CR42] Porter RJ, Bowie CR, Jordan J, Malhi GS (2013). Cognitive remediation as a treatment for major depression: A rationale, review of evidence and recommendations for future research. Australian and New Zealand Journal of Psychiatry.

[CR43] Savaskan E, Müller SE, Böhringer A, Schulz A, Schächinger H (2008). Antidepressive therapy with escitalopram improves mood, cognitive symptoms, and identity memory for angry faces in elderly depressed patients. International Journal of Neuropsychopharmacology.

[CR44] Sawilowsky SS (2009). New effect size rules of thumb. Journal of Modern Applied Statistical Methods.

[CR45] Saxena S, Chandiramani K, Bhargava R (1998). WHOQOL-Hindi: A questionnaire forassessing quality of life in health care settings in India. World Health Organization Quality of Life. National Medical Journal of India.

[CR46] Sheehan, D. V. (2016). *M.I.N.I. Mini International Neuropsychiatric Interview English Version 7.0.2 for DSM-5.* https://www.harmresearch.org/index.php/mini-international-neuropsychiatric-interviewmini/

[CR47] Singh AK (1998). Tests, Measurements, and Research Methods in Behavioural Sciences.

[CR48] Singh J, Singh S, Chavan BS, Gupta S, Arun P, Kaur D, Kaur N, Sharma A (2023). Efficacy of cognitive training program given to patients with schizophrenia using computer tablets: A preliminary study. International Journal of Cognitive Therapy.

[CR49] Skandali N, Rowe JB, Voon V, Deakin JB, Cardinal RN, Cormack F, Passamonti L, Bevan-Jones WR, Regenthal R, Chamberlain SR, Robbins TW, Sahakian BJ (2018). Dissociable effects of acute SSRI (escitalopram) on executive, learning and emotional functions in healthy humans. Neuropsychopharmacol.

[CR50] Smarr KL, Keefer AL (2011). Depression inventory-II (BDI-II) centre for epidemiologic studies depression scale (CES-D), geriatric depression scale (GDS), hospital anxiety and depression scale (HADS), and patient health questionnaire-9 (PHQ-9). Arthritis Care and Research.

[CR51] Stevenson CS, Whitmont S, Bornholt L, Livesey D, Stevenson RJ (2002). A cognitive remediation programme for adults with attention deficit hyperactivity disorder. Australian and New Zealand Journal of Psychiatry.

[CR52] Tajrishi, K. Z., Mohammadkhani, S., & Jadidi, F. (2011). Metacognitive beliefs and negative emotions. *Procedia – Social and Behavioral Sciences, 30*, 530–533. 10.1016/j.sbspro.2011.10.103

[CR53] Tomás P, Fuentes I, Roder V, Ruiz JC (2010). Cognitive rehabilitation programs in schizophrenia: Current status and perspectives. International Journal of Psychology and Psychological Therapy.

[CR54] Üstün TB, Ayuso-Mateos JL, Chatterji S, Mathers C, Murray CJL (2004). Global burden of depressive disorders in the year 2000. The British Journal of Psychiatry.

[CR55] Vatnaland T, Vatnaland J, Friis S, Opjordsmoen S (2007). Are GAF scores reliable in routine clinical use?. Acta Psychiatrica Scandinavica.

[CR56] Wang Y-P, Gorenstein C (2013). Psychometric properties of the Beck depression inventory-II: A comprehensive review. Brazilian Journal of Psychiatry.

[CR57] Wells A (2009). Metacognitive therapy for anxiety and depression.

[CR58] Wells A, Cartwright-Hatton S (2004). A short form of the metacognitions questionnaire: Properties of the MCQ-30. Behavior Research and Therapy.

[CR59] Williams JR (2008). The declaration of Helsinki and public health. Bulletin of the World Health Organization.

[CR60] Woolf C, Lampit A, Shahnawaz Z, Sabates J, Norrie LM, Burke D, Naismith SL, Mowszowski L (2022). A systematic review and meta-analysis of cognitive training in adults with major depressive disorder. Neuropsychology Review.

[CR61] World Health Organization (1982). The ICD-10 classification of mental and behavioural disorders: clinical descriptions and diagnostic guidelines. https://www.who.int/classifications/icd/en/bluebook.pdf. Accessed 15 Jul 2020.

[CR62] World Health Organization (2001). The world health report 2001: Chapter four mental health policy and service provision. https://www.who.int/whr/2001/en/whr01_ch4_en.pdf?ua=1. Accessed 25 Jul 2020.

[CR63] World Health Organization (2017). Depression and other common mental disorders: global health estimates. https://apps.who.int/iris/bitstream/handle/10665/254610/WHO-MSD-MER-2017.2-eng.pdf;jsessionid=0760CF55F9F8FDCCB0582BEFE1877339?sequence=1. Accessed 1 Aug 2020.

