# Correction: Effect of cognitive retraining treatment in mild to moderate depressive disorders

**DOI:** 10.1186/s41155-023-00275-x

**Published:** 2023-10-23

**Authors:** Aarzoo Gupta, Santha Kumari

**Affiliations:** 1 Department of Psychiatry, Government Medical College & Hospital, Chandigarh, India; 2 School of Humanities & Social Sciences, Thapar Institute of Engineering & Technology, Patiala, India

**Correction: Psicologia: Reflexão e Crítica 36, 28 (2023)**

**https://doi.org/10.1186/s41155-023-00269-9**

Following publication of the original article (Gupta & Kumari, 2023), the editor reported two errors in the article:

The abstract has been added; the author’s name Aarzoo has been corrected to Aarzoo Gupta

The original article (Gupta & Kumari, 2023) has been updated.
